# Ontology-Enriched Specifications Enabling Findable, Accessible, Interoperable, and Reusable Marine Metagenomic Datasets in Cyberinfrastructure Systems

**DOI:** 10.3389/fmicb.2021.765268

**Published:** 2021-12-08

**Authors:** Kai L. Blumberg, Alise J. Ponsero, Matthew Bomhoff, Elisha M. Wood-Charlson, Edward F. DeLong, Bonnie L. Hurwitz

**Affiliations:** ^1^Department of Biosystems Engineering, University of Arizona, Tucson, AZ, United States; ^2^E.O. Lawrence Berkeley National Laboratory, Environmental Genomics and Systems Biology Division, Berkeley, CA, United States; ^3^Daniel K. Inouye Center for Microbial Oceanography, University of Hawai‘i, Honolulu, HI, United States; ^4^BIO5 Institute, University of Arizona, Tucson, AZ, United States

**Keywords:** ontology, FAIR, metagenomics, marine microbiology, cyberinfrastructure (CI), next generation sequencing—NGS, omics

## Abstract

Marine microbial ecology requires the systematic comparison of biogeochemical and sequence data to analyze environmental influences on the distribution and variability of microbial communities. With ever-increasing quantities of metagenomic data, there is a growing need to make datasets Findable, Accessible, Interoperable, and Reusable (FAIR) across diverse ecosystems. FAIR data is essential to developing analytical frameworks that integrate microbiological, genomic, ecological, oceanographic, and computational methods. Although community standards defining the minimal metadata required to accompany sequence data exist, they haven’t been consistently used across projects, precluding interoperability. Moreover, these data are not machine-actionable or discoverable by cyberinfrastructure systems. By making ‘omic and physicochemical datasets FAIR to machine systems, we can enable sequence data discovery and reuse based on machine-readable descriptions of environments or physicochemical gradients. In this work, we developed a novel technical specification for dataset encapsulation for the FAIR reuse of marine metagenomic and physicochemical datasets within cyberinfrastructure systems. This includes using Frictionless Data Packages enriched with terminology from environmental and life-science ontologies to annotate measured variables, their units, and the measurement devices used. This approach was implemented in Planet Microbe, a cyberinfrastructure platform and marine metagenomic web-portal. Here, we discuss the data properties built into the specification to make global ocean datasets FAIR within the Planet Microbe portal. We additionally discuss the selection of, and contributions to marine-science ontologies used within the specification. Finally, we use the system to discover data by which to answer various biological questions about environments, physicochemical gradients, and microbial communities in meta-analyses. This work represents a future direction in marine metagenomic research by proposing a specification for FAIR dataset encapsulation that, if adopted within cyberinfrastructure systems, would automate the discovery, exchange, and re-use of data needed to answer broader reaching questions than originally intended.

## Introduction

Recently, efforts have been made to encourage scientific data producers and publishers to make their data Findable, Accessible, Reusable, and Interoperable (FAIR) (Wilkinson et al., 2016), referred to as the FAIR guiding principles for scientific data management and stewardship. These principles provide high-level suggestions for how to improve the digital ecosystem of data producers, publishers, and consumers (Wilkinson et al., 2016). The FAIR principles state that datasets should: (1) be annotated with metadata to allow for the discovery of datasets based on their metadata (Findability), (2) be freely accessible using standard protocols (Accessibility), (3) use standardized metadata that are able to work together in combination with other metadata (Interoperability), and (4) employ metadata which can thoroughly describe a plurality of accurate and relevant attributes from the dataset (Reusability). The authors specify that data should be FAIR, not only apply to humans but more importantly to automated web-discovery systems (referred to as machine-agents) (Wilkinson et al., 2016). The long-term objectives laid out by the authors of the FAIR principles are to facilitate the integration and reuse of published data to enable novel discoveries and innovations (Wilkinson et al., 2016). Long term ecological monitoring sites have data that are important for understanding the global ocean, however, these datasets (including metagenomic data) are often not consistently made available in a manner following using the FAIR data principles.

Advances in sequencing technologies have enabled the generation of unprecedented volumes of metagenomic data (Mitchell et al., 2016). This has resulted in a proliferation of publicly available metagenomic datasets, especially from marine sampling expeditions which have enabled novel insights into the taxonomic structure and functional capabilities of microbial communities from diverse ocean environments (Karl and Lukas, 1996; Rusch et al., 2007; Zinger et al., 2011; Sunagawa et al., 2015; Zheng et al., 2016; Mende et al., 2017; Biller et al., 2018). Better understanding the role of microbes in heterogeneous and dynamic marine ecosystems will require both longitudinal and temporal collections of large-scale and historical data. Therefore, these data will need to be integrated with one another, as well as linked with supplemental data detailing their broader physio-chemical context. Data will need not only to be made reusable within projects generated within a greater sampling campaign, but also across datasets generated by different campaigns as well as investigators.

Despite the proliferation of marine metagenomic and accompanying contextual data, the lack of commonly used FAIR principles by which to standardize the wide range of physicochemical attributes remains a barrier toward elucidating the biogeochemical and environmental drivers of community structure. Thus, efforts to compare across studies using meta-analyses have infrequently been attempted due to lack of interoperable data (Field et al., 2009). Further, as identified by the EarthCube Geoscience 2020 report, understanding critical changes to ecosystems requires appropriate historical context by which to detect long-term trends; a common need across geoscience domains^1^. By integrating disparate data types, such as ‘omics and environmental data, across many sampling efforts to cover larger spatiotemporal ranges, we can begin to fill the contextual void that currently limits our understanding of ecosystem resilience. By applying the FAIR data principles to these ‘omics datasets we can begin to realize this potential.

Today, metagenomic datasets are typically published within publicly available International Nucleotide Sequence Database Collaboration (INSDC) repositories such as the European Nucleotide Archive (ENA) and the National Center for Biotechnological Information (NCBI) (Nakamura et al., 2013). Thus, published metagenomic data are Findable, and after an optional holding period become Accessible as well. Additionally, the Genomic Standards Consortium (GSC) was established to standardize contextual data (a.k.a. metadata) requirements for genomic and metagenomic data (Yilmaz et al., 2011a). As a result of GSC efforts, several Minimum Information about any (x) Sequence (MIxS) checklists were created to establish a unified standard for the description of data commonly collected along with sequencing data (Yilmaz et al., 2011b). Contextual information about the environmental source, spatiotemporal location, and other environmental characteristics are essential to interpreting and analyzing metagenomic data (Yilmaz et al., 2011a). As such, the MIxS specifications are organized into a collection of environment-specific packages (e.g., water, soil), with discipline-specific metadata requirements (Yilmaz et al., 2011b).

In an effort to standardize their genomic and metagenomic contextual data, both the ENA and NCBI have integrated the MIxS checklists into their submission procedures (Karsch-Mizrachi et al., 2018; Mitchell et al., 2018); however, many metadata fields are not associated with ontology terms, thus hindering their Interoperability. Additionally, these datasets are not automatically reusable because MIxS metadata fields are entered as free text. Although the MIxS checklists provide a syntax for how data should be entered (as well as examples), users are expected to correctly type out ontology term identifiers and labels or provide both numeric values and free text units. Finally, there are no mechanisms to ensure data conforms to the expected data type and syntax (e.g., checks if a number is provided, if units are provided, or if a correct ontology term is provided). So, although there is more widespread use of the MIxS checklists in major genomic sequencing repositories, the resulting physicochemical data accompanying the genomic data sequences remains inconsistent.

To perform meta-analyses, it is not only important for datasets to use consistent vocabularies, but it’s also important for those vocabularies to follow a rigorous semantic framework that enables computation and discovery. This can be accomplished by utilizing vocabularies which serve as the metadata conformant to the FAIR data principles (Wilkinson et al., 2016). All MIxS checklists mandate the use of terminology from a semantic resource called the Environment Ontology (ENVO) (Buttigieg et al., 2013, Buttigieg et al., 2016) for the annotation of the broad-scale environmental context, local-scale environmental context, and environmental medium of metagenomic data (Yilmaz et al., 2011b). ENVO provides semantic descriptions of environment types, environmental materials, and biomes, by which to annotate biological samples (Buttigieg et al., 2013, Buttigieg et al., 2016). Environmental information represented within ENVO is both human and machine-readable, hierarchically structured, and contains logical and machine-processable relationships to other represented entities. Such features make ENVO an ontology, rather than a controlled vocabulary (Buttigieg et al., 2016).

ENVO is not a standalone resource but is rather part of a larger consortium of ontologies called the Open Biomedical and Biological Ontologies (OBO) Foundry and Library (Smith et al., 2007). OBO ontologies use common design strategies to work together interoperably as a unified multidisciplinary knowledge representation model (Walls et al., 2014); each representing information from specific domains such as genomics via the Gene Ontology (GO) (Ashburner et al., 2000), and scientific investigative processes via the Ontology for Biomedical Investigations (OBI) (Bandrowski et al., 2016). In addition, ontologies are semantic web resources (Bechhofer et al., 2004), supporting querying features that enable the discovery of ontology terms based on input conditions (Prud’hommeaux and Seaborne, 2008). Ontologies also support reasoning, a process employing formal logic to determine the hierarchical placement of terms within an ontology, based on their relationships and logical statements (Kazakov et al., 2014). Due to their machine-readability and inclusion of machine-processable relationships between entities (Smith et al., 2007), ontologies are considered maximally robust semantic systems (McCreary, 2010).

Although ontologies are an important part of making data FAIR, simply annotating data with ontology terms is insufficient toward achieving the FAIR principles. Also required are standard practices for packaging, storing, and transferring datasets such that they can be made discoverable to machine agents via search routines on their ontology annotations. Other environmental science disciplines such as Oceanography and Meteorology achieve data interoperability by using the Network Common Data Form (NetCDF) standard (Rew and Davis, 1990; Brown et al., 1993). Although NetCDF has been used in genome-wide association studies based on precomputed Single Nucleotide Polymorphisms (Muñiz Fernandez et al., 2012), it is not intended to link genomic data to other sources such as physicochemical data.

Currently, a variety of web-based portals and cyberinfrastructure systems exist for metagenomic data. The Genomes Online Database (GOLD) hosted by the Joint Genome Institute (JGI) is an open access online portal that maintains contextual metadata associated with genomic and metagenome projects (Kyrpides, 1999; Mukherjee et al., 2019). GOLD makes use of the MIxS checklists to ensure that metagenomic datasets are consistent prior to analysis within the Integrated Microbial Genomes (IMG) system (Markowitz et al., 2007; Mukherjee et al., 2019). Another metagenomic portal is the widely used Meta Genomics Rapid Annotation using Subsystems Technology (MG-RAST) server, which also leverages the MIxS checklists and previous versions of ENVO (Meyer et al., 2008). The MGnify resource, hosted by the European Bioinformatics Institute (EBI), is another metagenomic analysis suite that aggregates metagenomic data from ENA (Mitchell et al., 2018), also linking to metadata sourced from MIxS checklists. Finally, a recent United States Department of Energy (DOE) initiative, the National Microbiome Data Collaborative (NMDC) was launched to support the data science ecosystem as well as community practices around making ‘omics data FAIR (Eloe-Fadrosh et al., 2021; Vangay et al., 2021). Although these resources do leverage existing standards such as MIxS checklists, there are currently not commonly accepted workflows for connecting metagenomic data with accompanying physicochemical data to make them FAIR implemented within existing metagenomic web portals.

In their 2017 work Wilkinson et al. (2017) demonstrated an example of how several web and semantic web technologies can be brought together to create a reference implementation of an interoperability architecture to enhance the discovery, integration, and reuse of biological data in accordance with the FAIR principles. To unlock the full potential of metagenomic data to elucidate the interactions between physicochemical gradients and the structure and function of microbial communities, a standardized containerization system analogous to NetCDF or that demonstrated in by Wilkinson et al. (2017) is needed. Such a system will need to (1) semantically annotate marine metagenomic and contextual data with ontologies, (2) containerize data products and their semantic annotations such that they make the data discoverable via its constituents to machine searches within cyberinfrastructure systems, and (3) support the transfer of containerized data products between cyberinfrastructure systems.

Here we demonstrate a prototype for FAIR re-use of metagenomic datasets using ontology-enriched specifications in Planet Microbe, a cyberinfrastructure system. Planet Microbe is the first data portal to offer FAIR data for large-scale analyses across multiple marine metagenomic studies (Ponsero et al., 2021). Current datasets encompass more than 2,300 samples collected from multiple projects around the world. These harmonized datasets fuel the search feature to allow users—including scientists, educators, and citizen scientists—to run computational tools on samples across systems. Planet Microbe is not a monolithic database and analysis system, nor a public data repository, but rather a collection of data products and tools that are grouped together under a common light-weight web-based interface. The approach to developing data packages we describe here is a way to package datasets as products in and of themselves and can be used by any person or system independently. In our working example at Planet Microbe, these data are containers that can be ported from system-to-system or cloud-to-cloud. Thus, all data packages and tools developed here are independent resources for the community. Moreover, the approach we propose is a foundation and set of standards that can be developed by the community over time, building upon and extending the data products currently available in Planet Microbe.

## Results and Discussion

### Open Biomedical and Biological Ontologies-Frictionless Data Products

**FIGURE 1:**
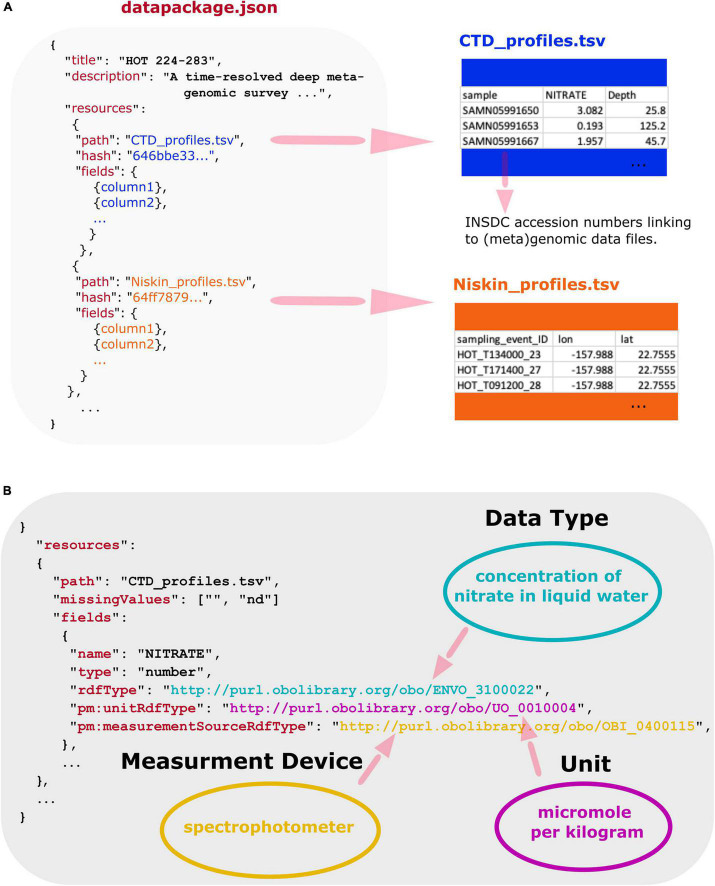
**(A)** Overview of Planet Microbe Frictionless Data Packages that for any given project includes a “datapackage.json” file containing machine-readable descriptive information about the individual resource TSV files and their columns, as well as the original (or slightly modified) data files. Note that at least one data file per Data Package also includes data columns with International Nucleotide Sequence Database Collaboration (INSDC) accession numbers linking to (meta)genomic datasets. The machine readable JSON description enables data to be exposed to and processed by machine systems. Additionally, the JSON files contain information about the resource file path and md5 checksums (hashes) allowing for complete transferability of multi-component datasets between systems. **(B)** Simplified view of data annotations used to markup a dataset column in a Planet Microbe Frictionless Data Package for an individual data column from an example data set resource. Multiple reusability indicators are built into the system. This includes missing values checklists that describe datasets-specific strings specifying “NA” values or missing data. Additionally, the expected data type (e.g., “number”) is specified and can be used to validate the data to make sure it is as expected (e.g., a numeric value not a string). Unit interoperability is specified by the pm:unitRdfType, annotating the units of measurement with a term [e.g., UO “*micromole per kilogram*” (UO:0010004)] from the Units Ontology. Collection instrumentation is specified by a term from the Ontology for Biomedical Investigations in the pm:measurmentSourceRdfType [e.g., OBI “*spectrophotometer*” (OBI:0400115)] to denote the type of device with which the measurement was taken. Finally, attribute types are made discoverable to machine systems via the rdfType, such as ENVO “*concentration of nitrate in liquid water*” (ENVO:3100022), specifying what the data attribute is about.

Using a combination of Frictionless Data and OBO ontologies, we encapsulate multi-part marine metagenomic datasets and their accompanying physicochemical and environmental contextual data into FAIR data products. The Planet Microbe Data Package specification is built upon Frictionless Data^2^. Frictionless Data is a technical standard for the containerization, publication, and mobilization of data. Frictionless Data provides specifications and software libraries for the construction and use of Frictionless Data Packages, including software tools for loading Data Packages into database systems. Frictionless Data Packages are JavaScript Object Notation Format (JSON) files in which metadata about multiple data resources such as tab-separated value (TSV) files can be encapsulated (see Figure 1A). Annotation metadata, such as ontology terms can be added within Frictionless Data Package JSON files to describe the resource files. The Data Packages enable comprehensive data validation and annotation. See Figure 1B for an example of this in which a single column from a dataset TSV file is annotated with a triad of OBO ontology terms to capture (1) what the data column is about, (2) the units of measure the data is reported in, and (3) the measurement device used to collect the data. When used together in systems like the Planet Microbe cyberinfrastructure web portal, the Data Packages can enable novel queries across integrated data sources. See the section on addressing biological questions. Additionally, the Data Packages can be used independently of the Planet Microbe portal, by anyone for any purpose. The Data Packages are available in github.^3^

### Planet Microbe Implementation

In Planet Microbe, we implemented the following FAIR data properties to which the Planet Microbe OBO-Frictionless Data Package specification should conform to ameliorate the FAIR use of marine metagenomic data within cyberinfrastructure systems. We define the proposed properties as follows: (1) Machine-exposability: the ability for containerized data products to be unpacked into, understood by, and used within various cyberinfrastructure systems; (2) Complete transferability: the ability for multi-component datasets to be easily exchanged between informatic systems employed by data producers and consumers; (3) Reusability indicators: comprehensive data annotations including data type checks and machine-readable semantics enabling additional decisions to be made about whether or not data attributes should be reused in combination; and (4) Attribute-discoverability: the ability to discover individual constituents from datasets based upon their annotation with machine-readable semantics specifying the type of the attribute.

#### Machine-Exposability

Although not prescribing a specific solution to making data FAIR, the FAIR Principles emphasize machine-actionability, the ability for computational systems to find, access, interoperate, and reuse data in an automated manner. The current standard for annotating attributes accompanying metagenomic datasets, the MIxS checklists, are not machine-readable. Additionally, there is no standard way to encapsulate marine metagenomic datasets along with their annotation semantics within a machine-readable framework. Thus, to make marine metagenomic data FAIR, we developed an informatic system that can connect machine-readable annotation semantics for both data attributes and provenance information, with data. The ability for Frictionless Data Packages to enable the machine-readable annotation of variable resources, is highly amenable for the management of marine metagenomic datasets, which often contain multiple components each with a variable template.

#### Complete Transferability

Another consideration when designing the Planet Microbe Frictionless Data Package system was what we refer to as the property complete transferability, the ability to completely exchange multi-component data products between data producers and consumers. Complete transferability is also satisfied using Frictionless Data Packages, which enable multiple data resources to be described within a master metadata resource. All files including the master JSON file and resource files can be stored together within a common directory structure where the relative paths within the directory to the resource files are described within the master JSON file. The master JSON file contains the MD5 checksums of the resource files, thus the complete transferability of all data files can be machine-validated by checking if all the resource files exist where they should and if they have the correct MD5 checksum.

#### Reusability Indicators

For datasets to be reused in meta-analyses, it may be necessary to perform certain consistency checks or make decisions about whether or not data attributes should be reused in combination. We refer to these properties as reusability indicators and have incorporated three of them into the Planet Microbe Frictionless Data Packages. These include checks for (1) data inconsistencies, (2) unit interoperability: machine-readable semantics specifying the units attributes were measured in, and (3) measurement device indicators, semantics specifying what devices were used to measure data attributes.

##### Checks for Inconsistencies

Before data can be reused, we must ensure that it is reported in a way that is consistent with its intended use and purpose. Frictionless Data Packages enable this by allowing for data annotation at a fine level of resolution, namely specifying the expected type of each attribute from an original data source to be “string,” “number,” or “datetime.” In addition, Frictionless Data provides software libraries for data validation, making sure it conforms to these user-specified constraints. Users can further specify expected formats to which data columns are expected to conform to, for example, specifying a column of collection dates to be of type “datetime,” which follow a specific format such as “%Y-%m-%dT%H:%M:%SZ.” These Frictionless Data validation features were leveraged by scripts we developed to validate the Planet Microbe Data Packages. This enabled us to ensure the original data sources were formatted correctly, while preserving the data’s original configuration (e.g., datetime formatting). During validation of the original datasets a reasonable number of inconsistencies were found and were logged in the “README.md” files associated with each Data Package. There also exists community software tooling created by users within the Frictionless Data community, such as the Good Tables python library and command line tool for validating and transforming tabular data within Frictionless Data Packages (Heughebaert, 2020).^4^ In addition to our own validation script, we used the Good Tables validation software to add numeric range constraint checks for latitude and longitude values in the Planet Microbe Data Packages. We did so by adding constraint code blocks to fields (parameters) within Planet Microbe Frictionless Data Packages to specify minimum and maximum acceptable values (e.g.,–90 to 90 and –180 to 180 for latitude and longitude, respectively). This provides an additional sanity check on the data and helps catch errors such as swapped latitude and longitude values. See Supplementary Figure 1 showing an example of a Planet Microbe Data Package with range constraints for a latitude field from the Beyster Family Fund and Life Technologies Foundation-funded Global Ocean Sampling Expedition (GOS), 2009–2011 dataset. By building these types of consistency checks into the Planet Microbe Data Packages, we were able to future proof the data, protecting ourselves and future reusers of these data products against inconsistencies.

##### Unit Interoperability

Another key attribute required for data to work interoperably is that the units of measure in which individual data attributes were collected, a concept such as “nanograms per liter” must be understandable at a machine level. Although the MIxS checklist defines standard units in which ‘omic accompanying data should be reported in, conformance cannot just be assumed. Thus, within the Planet Microbe Frictionless Data Package specification, semantic annotations for unit types are included. Many unit vocabularies and systems exist which would be fit for this purpose (Madin et al., 2007; Rijgersberg et al., 2011; FAIRsharing Team, 2015). Here we opted to make use of terminology from the Units Ontology (UO) (Gkoutos et al., 2012) for the units of measure annotations included in the Frictionless Data Package JSON files. This was done both to remain within the OBO knowledge modeling paradigm, as well as for the ease of use in importing UO, within the PMO application ontology, as UO is an OBO ontology following standard formatting conventions. See Figure 1 for an example of an individual parameter within a Planet Microbe Data Package annotated with a UO unit term.

##### Measurement Device Terms

Another reusability indicator, which further enriches the descriptive value of annotated data, is the inclusion of machine-readable semantics describing the instrumentation used in the collection of individual data attributes. A variety of semantic systems for instrumentation exist such as the NERC P10, the Rolling Deck to Repository (R2R) device type vocabulary, and the Biological and Chemical Data Management Office (BCO-DMO)’s instruments vocabularies (Coburn et al., 2012; Chen et al., 2014; Moncoiffe and Kokkinaki, 2018). However, for the Planet Microbe Frictionless Data Package specification, we again opted to stay within the OBO paradigm for ease of use with the application ontology, and reused measurement device terminology from the OBO ontology OBI (the Ontology for Biomedical Investigations) (Bandrowski et al., 2016). Although the information about instrumentation was not as easy to discern from the original project data sources, several examples of annotations with OBI measurement devices terms have been included in the Planet Microbe Frictionless Data Packages. Information regarding the use of high-performance liquid chromatography instruments was most generally deciphered from the source repositories for the Planet Microbe Data Packages. As a result, 1593 annotations with “*high performance liquid chromatography instrument*” (OBI:0001057) were made. Other measurement devices for which a reasonable basis for annotation from the metadata in the source repositories included 9 measurement device annotations with the class “*flow cytometer*” (OBI:0400044), 11 annotations with “*fluorometer*” (OBI:0400143), 1 instance of the use of a “microscope” (OBI:0400169), and 7 clear uses of a “*spectrophotometer*” (OBI:0400115). See Figure 1B, for an example of an individual parameter from a Planet Microbe Data Package annotated with an OBI measurement device term.

#### Attribute-Discoverability

The final property we built the Planet Microbe Frictionless Data Package specification to conform to we call attribute attribute-discoverability. By this we mean the ability for machine systems to search for data attributes based on their annotation types; types which should themselves be machine-readable semantics that exist within a larger knowledge representation framework.

##### Planet Microbe Application Ontology

To ensure attribute-discoverability of Planet Microbe Frictionless Data Packages, all semantic annotations used in the Data Packages are included within the Planet Microbe application (PMO).^5^ The PMO application ontology was built following OBO foundry tools^6^ (Ponsero et al., 2021) and includes ontology imports from other relevant OBO foundry ontologies such as ENVO, UO, and OBI. OBO ontologies are built following common design practices including shared top-level terms, relations annotations properties, and design patterns to ensure interoperability. Thus, when available, we leveraged appropriate terminology from these existing ontologies to maximize interoperability with external projects that also reuse those ontologies. However, not all spatiotemporal and physicochemical concepts needed for the annotation of marine metagenomic data were available from relevant OBO ontologies. Thus, within PMO we include additional terminology [e.g., “*latitude coordinate measurement datum*” “*start*” and “*stop*” (PMO:00000076) and (PMO:00000079), respectively] to more comprehensively annotate the datasets encapsulated within Planet Microbe Frictionless Data Packages. In addition, PMO includes terminology added to ENVO as a result of this work. See “Materials and Methods” sections on ENVO contributions, as well as Figure 1B for an example of an individual parameter within a Planet Microbe Data Package annotated with an ENVO chemical concentration term.

#### Limits of Semantic Harmonization

Although this work, when possible, included annotation information about the measurement devices used to collect data, this is not always clear from the source data repositories. Future data submission frameworks might consider mandating or more strongly encouraging the inclusion of information about measurement devices as part of the data submission process. This could be facilitated by automated protocols by instrument producers to capture structured and semantically enhanced data. Clearly annotating these types of differences in measurement devices would enable future systems and users to make decisions about what data can be compared based on the methodology used. Even if the data are about the same measurement or object (e.g., “chlorophyll a concentration”), the process by which these measurements were collected may result in non-comparable data. Furthermore, it should be noted that the precision of a concept being annotated is also important. It is more difficult to compare measurements annotated with a more generic concept such as “*concentration of chlorophyll in liquid water*” (ENVO:3100036), than data annotated with a more precise concept such as “*concentration of chlorophyll a in liquid water*” (ENVO:3100008). While the latter is specific enough to refer to a particular molecule, the former could be used to refer more generally to any mixture of chlorophyll compounds. For example, the “CHLPIG” parameter from some the Hawaiian Ocean Time Series (HOT) datasets is fluorometrically collected “chloropigment” data. As there are a variety of pigments associated with chlorophyll or “chloropigments,” we annotated this data with the more general term “*concentration of chlorophyll in liquid water*” (ENVO:3100036). Depending on the wavelengths used in the analysis protocol this parameter could be measuring a different set of compounds than other projects also reporting chlorophyll data using fluorometric methods. In such cases reporting the data type, unit type, and measurement device may not be sufficient to make data intercomparable. Additional efforts to intercalibrate methods and link protocols, perhaps using systems like Protocols.io, are also required.

### Addressing Biological Questions Using Harmonized Data

The second section of this paper addresses a series of biological queries of data integrated using the newly proposed specifications to demonstrate the efficacy of validating results against known biological and biogeochemical relationships and distributions.

#### Revisiting the Redfield Ratio

**FIGURE 2:**
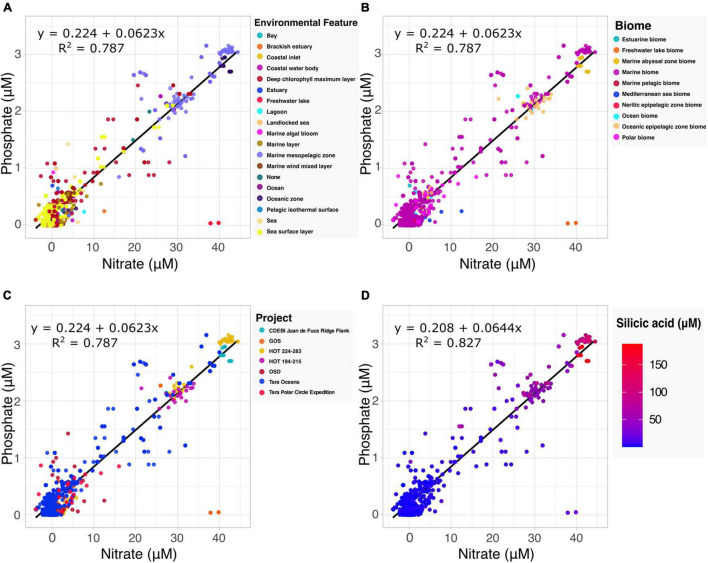
Examination of the Redfield ratio, the relationship between phosphate and nitrate concentrations, faceted by the various harmonized metadata types included in the Planet Microbe Data Packages. **(A)** The Redfield ratio colored by the 18 ENVO environmental feature types [e.g., “*bay*” (ENVO:00000032)]. **(B)** The Redfield ratio colored by the 10 ENVO biome types [e.g., “*estuarine biome*” (ENVO:01000020)]. **(C)** The Redfield ratio colored by the project name for the seven projects that include both phosphate and nitrate data. **(D)** The correlation between silicic acid and the Redfield ratio for a subset of the data shown in **(A–C)** that also include silicate data. Linear equations modeling phosphate as a function of nitrate and *R*^2^ are displayed in the panels. Nitrate, phosphate, and silicic acid are reported in micromolar concentrations.

The Redfield ratio describes the stoichiometric carbon to nitrogen to phosphate ratio occurring in marine phytoplankton and is fundamental to our understanding of marine biogeochemistry (Tyrrell, 2019). Here we explore how the proposed specification method can automate the discovery of data by which to build upon existing hypotheses. Drawing upon the harmonized data and accompanying metadata provided by the Planet Microbe Data Packages, we selected 1076 samples with both nitrate and phosphate values (Figures 2A–C). Performing a linear regression on the selected data, we found the coefficient for the phosphate to nitrate ratio to be 0.0623. This is very close to 0.0625, the inverse of the 16:1 nitrate to phosphate ratio reported by Redfield (1934) and reconfirmed in many follow up studies (Takahashi et al., 1985; Anderson and Sarmiento, 1994).

The source data (following the MIxS checklists) contains accompanying metadata about the environmental context of the samples. Specifically, “*biome*” (ENVO:00000428), “*environmental feature*” (ENVO:00002297) and “*environmental material*” (ENVO:00010483) terms from ENVO. This metadata, which was cleaned and harmonized within the Planet Microbe Data Packages can be used to provide additional views into the data (Figures 2A,B). For example, there are two observations at approximately 37–40 μM nitrate and 0 μM phosphate that clearly deviate from the Redfield ratio. This can be explained by the ENVO environmental feature and biome annotations of these samples which were “*freshwater lake*” (ENVO:00000021) and “*freshwater lake biome*” (ENVO:01000252), respectively. This is consistent with the fact that, unlike pristine marine systems, freshwater systems (especially those experiencing anthropogenic influence) are not necessarily expected to follow the Redfield ratio (They et al., 2017).

Investigating the correlation between additional parameters and the Redfield ratio, we also searched for data with nitrate, phosphate, and silicic acid values within the Planet Microbe Data Packages. This narrowed the original dataset down from 1076 to 1063 samples. We used these 1063 samples, as well as the original 1076 samples with depth values to investigate the correlation between the Redfield ratio with silicic acid and depth. We found that silicic acid and depth have moderate positive correlations with the nitrate to phosphate ratio, with Spearman correlations of 0.420 and 0.413, respectively (*p*-values less than 2.2e-16). The individual Spearman correlations between phosphate and nitrate with silicic acid are higher, being 0.677 and 0.695, respectively (*p*-values less than 2.2e-16). See Figure 2D showing that the observed silicic acid concentration varies proportionally with increased nitrate and phosphate concentrations. This is consistent with the fact that diatoms, which require silica for their cell walls, can be an important source of sinking particulate organic matter and nutrient export.

#### Latitudinal Variation Across Environments and Physicochemical Gradients

**FIGURE 3:**
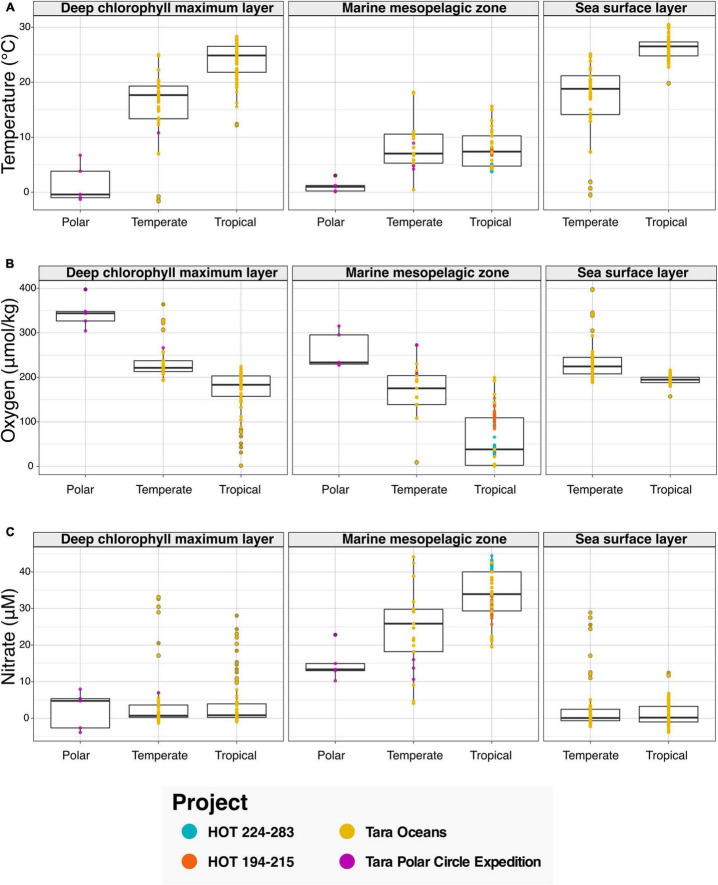
Bar graphs showing physicochemical parameters faceted by annotation with various ENVO “*environmental feature*” (ENVO:00002297) terms serving as descriptors of environments. Data in this analysis are derived from various HOT and Tara projects that are annotated with the following ENVO terms: “*deep chlorophyll maximum layer*” (ENVO:01000326), “*marine mesopelagic zone*” (ENVO:00000213), and “*sea surface layer*” (ENVO:01001581). Additionally, the data are binned by latitude values into major climatic conditions “*polar*” (ENVO:01000238), “*temperate*” (ENVO:01000206), and “*tropical*” (ENVO:01000204). **(A–C)** The ENVO parameters: “*temperature of water*” (ENVO:09200014), “*concentration of dioxygen in liquid water*” (ENVO:3100011), and “*concentration of nitrate in liquid water*” (ENVO:3100022) in units of degree Celsius, and micromolar, respectively.

We then leveraged the newly harmonized spatiotemporal and physicochemical variables, in combination with the descriptions of environment types provided with sequence data (see Figure 3). To showcase the discoveries that can result from the integration of the aforementioned data types, we asked the question “What variations in physicochemical parameters do we observe across environmental types and climate zones?” To examine this question, we searched the Planet Microbe Data Packages for data which included several physicochemical parameters including temperature, pH, and oxygen and nitrate concentrations. We analyzed 995 samples with temperature oxygen and nitrate values (Figure 3), and 488 samples with pH values (Supplementary Figure 2).

Results show that as expected, temperature increases in the “*deep chlorophyll maximum layer*” (ENVO:01000326) and “*sea surface layer*” (ENVO:01001581) environments between polar, temperate, and tropical zones (Figure 3A). Additionally, there is not a significant difference between the temperature of temperate and tropical zones in the “*marine mesopelagic zone*” (ENVO:00000213). This result is consistent with the description of the mesopelagic zone being the uppermost region of a pelagic aphotic zone, bounded at the thermocline temperature transition zone (del Giorgio and Duarte, 2002)^7^. Although these results regarding the variation in temperature across environment types and climatic zones are not new per say, they help to sanity check that this system is working correctly by providing expected results.

In addition to temperature, other physicochemical factors such as oxygen and nitrate have been used to identify and differentiate distinct marine environments (Sayre et al., 2017; Sutton et al., 2017). Examining the results for oxygen, we see decreases in oxygen concentrations between polar, temperate, and tropical zones in all environment types (Figure 3B). It is notable that although the dissolved oxygen values for the “*deep chlorophyll maximum layer*” (ENVO:01000326) and “*sea surface layer*” (ENVO:01001581) are similar across temperate and tropical zones, there is a large ∼150 μM difference between the averages of temperate and tropical samples oxygen values in the analyzed “*marine mesopelagic zone*” (ENVO:00000213) samples. Many of the low oxygen mesopelagic tropical samples are sourced from 500 to 1,000 meter depths from the subtropical oceans near Hawai‘i. This is consistent with the known oxygen minimum found at ∼800 m at Station ALOHA (Bingham and Lukas, 1996), as well as previous findings that low oxygen water masses are common in midwater depth range of 500–1,500 m in subtropical regions, due to the microbial remineralization of sinking organic matter (Jürgens and Taylor, 2018).

Analyzing the results for nitrate, we observe that the average of nitrate values in the “*marine mesopelagic zone*” (ENVO:00000213) are quite different from the other environment types (Figure 3C). We observe a similar pattern with pH values in tropical samples being much lower in the “*marine mesopelagic zone*” (ENVO:00000213) than in the “*sea surface layer*” (ENVO:01001581) (Supplementary Figure 2). It is notable that for the environmental descriptor “*marine mesopelagic zone*” (ENVO:00000213), we observe differences in the values of three physiological parameters: temperature, nitrate, and pH in tropical samples relative to the other ENVO environment types. This serves as an example of the expert information about specific concepts captured within an ontology (e.g., environment types in ENVO) being differentiable based on observed patterns in real world data.

#### Harmonization Across Time Series

**FIGURE 4:**
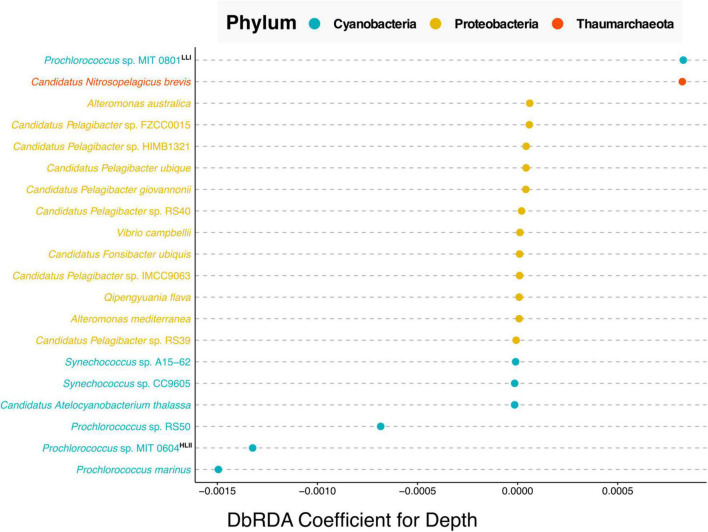
Cleveland’s dot plot showing the top 20 coefficients ordered by absolute value, derived from a distance-based redundancy analysis (dbRDA) of the relative abundance of bacterial and archaeal species against depth. Species are colored by taxonomic phylum with Cyanobacteria in blue, Proteobacteria in yellow and Thaumarchaeota in orange. LLI and HLII indicate “high light adapted” and “low light adapted,” respectively based on the analysis of Biller et al. (2014). The depth of samples used in this analysis ranged between 25 and 125 m.

Finally, we explored the benefits derived from data harmonization across time series. The Hawaiian Ocean Time Series (HOT) study is one of the longest running open ocean time-series surveys spanning more than 30 years of data (Karl and Church, 2014). Resulting from this work’s data harmonization we were able to combine existing data from two separate HOT metagenomic projects one that used pyrosequencing (Bryant et al., 2016), and a second that leveraged Illumina sequencing (Mende et al., 2017) into a new dataset comprising 52 metagenomic samples with 19 common physicochemical variables. Shown in Supplementary Figure 3 are the correlation coefficients between the 19 variables. Nine of the 19 variables have significant moderate correlations with depth (*p*-values less than 0.01 and Spearman correlation coefficients greater than 0.4). This along with the fact that depth is an important factor regulating the distribution of microbial species within marine systems, we performed a taxonomic analysis of those samples to investigate what microbial species vary most with depth in the North Pacific Subtropical Gyre (Figure 4).

The results of the dbRDA analysis shown in Figure 4, can be interpreted as follows, the coefficients indicate the extent to which the abundance of any given microbial species correlates with depth. Positive correlation values indicate species that increase in relative abundance with depth. Negative values indicate species that are anti-correlated with depth, i.e., species whose relative abundance decreases with depth. Near-zero values indicate no correlation with depth. This analysis shows that in the North Pacific Subtropical Gyre, of the top twenty correlations of species with depth, the five strongest, non-zero correlations are Cyanobacteria and Thaumarchaeota species. Whereas the majority of non-zero correlations are from the phylum Proteobacteria.

Our results showed that the Cyanobacteria related to *Prochlorococcus* sp. MIT 0801, and Thaumarchaeota most closely affiliated with *Candidatus Nitrosopelagicus brevis*, were more abundant with increased depth. The former is consistent with the findings reported by Biller et al. (2014), where the authors performed a phylogenetic analysis of *Prochlorococcus* strains which they paired with physiological studies on isolate representatives from a subset of the species in the analysis. The authors’ results determined that the MIT 0801 strain is in a low light adapted *Prochlorococcus* subclade, thus validating our result that the MIT 0801 strain is correlated with depth as it prefers lower light levels found deeper in the water column (positive correlation value in Figure 4).

Regarding the Thaumarchaeota *Candidatus Nitrosopelagicus brevis*, our results show this archaeon to be most prevalent in upper water column samples from ∼125 m depth. Thaumarchaeota in general, and specifically *Candidatus Nitrosopelagicus brevis*, have been shown to be abundant microbes below ocean surface waters (Santoro et al., 2015). Furthermore, prior studies have shown that Thaumarchaeota begin to be detectable right around the deep chlorophyll maximum at ∼125 m at Station ALOHA (Mincer et al., 2007; Lincoln et al., 2014). Our results build upon these findings adding that *Candidatus Nitrosopelagicus brevis* are also likely low light adapted as they are most abundant at greater depths following the same pattern as the known low light adapted *Prochlorococcus* sp. MIT 0801 strain.

All three species most anti-correlated with depth are *Prochlorococcus* strains including *Prochlorococcus* sp. RS50, *Prochlorococcus* sp. MIT 0604, and *Prochlorococcus marinus*. The aforementioned work of Biller et al. (2014) classified the *Prochlorococcus* sp. MIT 0604 as belonging to a high light adapted clade, which is congruent with our results showing MIT 0604 to be more abundant at shallower depths.

Our results about the *Prochlorococcus* sp. MIT 0604 and *Prochlorococcus* sp. MIT 0801 ecotype depth distributions are consistent with a newly proposed *Prochlorococcus* taxonomy, in which the authors identified five *Prochlorococcus* genera with distinct ecological attributes (Tschoeke et al., 2020). According to the proposed model, the *Eurycoliumgenus* genus, including *Prochlorococcus* sp. MIT 0604 are associated with high temperature and oligotrophic environments, whereas the genera *Prolificoccus* including *Prochlorococcus* sp. MIT 0801 are most abundant at low temperatures (Walter et al., 2017; Tschoeke et al., 2020). This is supported in our results by a –0.570 spearman correlation between depth and temperature, see Supplementary Figure 3, hence the noted similarity in *Prochlorococcus* temperature distribution patterns.

Regarding *Prochlorococcus marinus*, it is known that at the strain level there are a variety of both high and low light adapted ecotypes (Rocap et al., 2003; Tschoeke et al., 2020). Although our analysis was only able to determine the *Prochlorococcus marinus* at a species level, it is notable that the *Prochlorococcus marinus* sequences detected were the most strongly anti-correlated with depth. This indicates that high light adapted *Prochlorococcus marinus* ecotypes are more abundant than low light adapted ecotypes in the North Pacific Subtropical Gyre, consistent with prior oceanographic studies (Johnson et al., 2006; Thompson et al., 2018).

A final result derived from this analysis concerns the depth distribution of *Prochlorococcus* sp. RS50. The RS50 genome was assembled in 2017 based on surface samples collected from the Red Sea in 2014 but has not been published upon.^8^ Currently very little is known about distribution and abundance of the *Prochlorococcus* sp. RS50 strain. Our results show that RS50 is one of the top three species most anti-correlated with depth, thus indicating that it is also high light adapted. This result demonstrates the utility of integrating and harmonizing metagenomic datasets to enable new discoveries from meta-analyses reusing previously published data. As updates are made to taxonomic databases (e.g., the inclusion of the RS50 genome), this will enable new results to be derived from reanalyzing published datasets together in meta-analyses.

## Materials and Methods

### Creating Frictionless Data Packages

We implemented the new OBO-Frictionless specification to create Frictionless Data Packages in Planet Microbe. These OBO-Frictionless Data Packages were created as follows, all data resources from a given project including resources linking to metagenomic datasets as well as constituent environmental contextual information was encapsulated within a master Frictionless Data Package JSON file. Inconsistencies in MIxS environmental contextual fields (e.g., “broad-scale environmental context”) were manually corrected. We also added new dataset columns containing links to the corresponding machine readable ENVO PURLs, e.g., “http://purl.obolibrary.org/obo/ENVO_01000253” for “freshwater river biome.” Data were standardized by a triad of OBO Foundry ontology terms to enable Interoperability of datasets: by (1) standardizing the data type annotation, (2) specifying machine-readable unit types that can be automatically converted into standardized or desired units programmatically, and (3) enabling users to further select which data should be compared in meta-analyses based on the measurement devices from which the data was collected. Additional dataset provenance information such as links to sources and usage licenses, as well as missing values, data types and data value constraints are also specified with the Frictionless Data Package JSON files. The Planet Microbe Frictionless Data Packages were validated using both the Good Tables python library and command line tool (Heughebaert, 2020; see text footnote 2), as well as custom scripts available from the planet microbe scripts repository.^9^ Planet Microbe Data Packages are available from the following repository (see text footnote 1). The protocol for generating Planet Microbe Data Packages is available from^10^.

### Environment Ontology Term Contributions

To standardize the representation of the large variety of physicochemical data attributes collected in accompaniment to the marine metagenomic datasets aggregated in Planet Microbe, new terminology was required. Addressing this issue in this work, we created a new ENVO module for chemical concentrations to create terms such as “*concentration of chlorophyll a in liquid water*” (ENVO:3100008). These new ENVO chemical concentration terms were created using a Dead Simple Ontology Design Pattern (DOSDP) (Osumi-Sutherland et al., 2017), in which input data in a tabular format is converted and compiled into Web Ontology Language (OWL) code. These new chemical concentration terms represent information about a chemical solvent, and material solute to machine systems by including an OWL equivalence axiom which links to terms from both from the Chemical Entities of Biological Interest (CHEBI) CHEBI ‘‘*chemical entity*’’ (CHEBI:24431) hierarchy for the former, and the ENVO ‘‘*environmental material*’’ (ENVO:00010483) hierarchy for the latter. For example, the equivalence class from the new ENVO term ‘‘*concentration of chlorophyll a in liquid water*’’ (ENVO:3100008) links it to both CHEBI ‘‘*chlorophyll a*’’ (CHEBI:18230) as well as ENVO ‘‘*liquid water*’’ (ENVO:00002006) in a machine searchable way. In addition, the equivalence axioms used in the new ENVO chemical concentration terms also include a linkage to the Phenotype and Trait Ontology (PATO) term ‘‘*concentration of*’’ (PATO:0000033). A total of 31 new chemical concentration terms were added to the new ENVO chemical concentration module^11^ as a result of this work.

### Measurement Device Annotation

In addition to semantic and unit types, we included the annotation of dataset parameters measurement device terms when possible. The following are examples of measurement device annotations that were included within the Planet Microbe Data Packages. Within the various HOT time series studies data about the concentration of chlorophyll a is reported twice using separate measurement devices. In one reported parameter the concentration of chlorophyll a is measured via a ‘‘*high performance liquid chromatography instrument*’’ (OBI:0001057) and in the other the data is measured using a ‘‘*fluorometer*’’ (OBI:0400143). Another example concerns organismal or cell count data, it is important to know if data were collected via flow cytometry vs. microscopy. Although it was possible to decipher various examples of cell counts which were measured using a ‘‘*flow cytometer*’’ (OBI:0400044) from the various Hawaiian Ocean Time Series projects as well as the Amazon continuum datasets. In contrast the single assignment to ‘‘microscope’’ (OBI:0400169) was made to the Ocean Sample Day (OSD) parameter: ‘‘Nanoplankton and microplankton aggregate’’ based on information in the OSD handbook.^12^ An example of a source ambiguity of employed measurement devices comes from the OSD project. Although OSD is well documented and used the microb3 vocabulary to annotate parameter types as well as their units, information about the measurement devices could only be found in the “Description” comments. Ambiguities in the handbook’s descriptions e.g., “Concentration of pigments (e.g., chlorophyll a) extracted and analyzed by fluorometry or HPLC” precluded the assignment of precise measurement type semantics.

### Whole Genome Sequencing Taxonomic Analysis

Analysis of Whole Genome Sequencing (WGS) data was conducted as follows, after downloading the fastq files deposited in the Short Read Archive (SRA) for the selected samples, a quality filtering and removal of human sequences was performed using fastqc v0.11.9 and trimGalore v0.6.6 with default parameters. Quality-filtered sequences were screened to remove human sequences using bowtie2 v2.4.2 against a non-redundant version of the Genome Reference Consortium Human Build 38, patch release 7.^13^ After quality control and human read filtering, metagenomes containing less than 10 million paired-end reads were discarded. Taxonomic profiling of the metagenomic samples was performed using the k-mer-based taxonomic classification software Kraken2 (Wood et al., 2019). Finally, the Kraken2 taxonomic abundances were reassigned to more specific taxonomic ranks using Braken (Lu et al., 2017). Briefly Kraken2 v2.1.1 was run on the paired read using the PlusPF database^14^ and Bracken v2.6.1 was run on the Kraken2 outputs. The code used for this analysis is available from the following repository.^15^

### Addressing Biological Questions

Data used to address various biological questions was collected using the Planet Microbe search interface^16^ and data download feature. Linear regression for Redfield ratios (Figure 2) was conducted using the “lm” R package and plotted using the “ggplot 2” package. Box plots for physicochemical variables (Figure 3 and Supplementary Figure 2) were plotted using the “ggplot 2” R package. The correlation heatmap (Supplementary Figure 3) was created using code sourced from the following repository,^17^ which leveraged the “corrplot” R library, using Spearman correlations as the method. Individual Spearman correlations of physicochemical variables to depth were done using the R “cor” package. Taxonomic profile data, generated by the WGS pipeline, was analyzed in R using the “phyloseq” and “microbiome” packages. A list of contaminant species was manually selected for removal prior to analysis. Shallow samples (under 100 k reads) were removed prior to normalization by relative abundance. Distance-based redundancy analysis (dbRDA) of relative taxonomic abundance against depth was performed using the “vegan” R package’s adonis method (Anderson, 2001), using Bray-Curtis dissimilarities. DbRDA results were plotted in a Cleveland’s dot plot (Figure 4) using the ggdotchart method from the ‘‘ggpubr’’ R package. Final versions of figures were edited with Inkscape.^18^

## Conclusion

Motivated by the long-term vision of harmonizing marine ‘omics and environmental data, in data products that are available to people and machines, we devised and implemented cyberinfrastructure specifications using OBO ontologies and Frictionless Data Packages to make data FAIR within the Planet Microbe web-portal. This new specification allows for marine ‘omic and contextual environmental data to be (1) exposed within machine searches, (2) be completely transferable between CI systems, (3) have mechanisms for automated data validation, (4) use common vocabularies for measurement types, devices and units, as well as (5) enable discovery of individual attributes from datasets based on their vocabulary annotations.

Furthermore, we leveraged this system to discover data with given oceanographic measurements, features, and thresholds to synthesize and analyze global datasets in novel ways. This work promotes a new understanding of the infrastructure and data coordination requirements for performing global ocean analyses at unprecedented spatial and temporal resolution, including the distribution of microbes and responses to environmental drivers. Taken together, Frictionless Data Packages in Planet Microbe provide a much-needed resource for uniting ‘omics data and associated data products from diverse ocean surveys with environmental data to allow the geosciences community to include federated oceanographic data in global models and analyses.

Finally, it should be noted that the cyberinfrastructure specifications presented here are not alone sufficient to ensure the reusability of existing and future global marine sampling projects. Importantly, future efforts will be needed to harmonize standard methods and protocols for dataset intercalibration.

## Data Availability Statement

Publicly available datasets were analyzed in this study. This data can be found here: https://github.com/hurwitzlab/planet-microbe-frictionless-data-package-paper.

## Author Contributions

KB wrote the manuscript, contributed to ontology development, annotated, assembled the Data Packages, and performed the statistical analyses. AP performed the WGS taxonomic analyses. MB developed the Planet Microbe scripts for Data Package validation and database integration. KB, AP, and MB developed the Planet Microbe Data Package specifications. AP, EW-C, ED, and BH contributed to the revision and preparation of the manuscript. BH, EW-C, and ED designed the project and scope of research. BH directed the project. All authors contributed to the article and approved the submitted version.

## Conflict of Interest

BH holds concurrent appointments as an Associate Professor of Biosystems Engineering at the University Arizona and as an Amazon Scholar. This publication describes work performed at the University Arizona and is not associated with Amazon. The remaining authors declare that the research was conducted in the absence of any commercial or financial relationships that could be construed as a potential conflict of interest.

## Publisher’s Note

All claims expressed in this article are solely those of the authors and do not necessarily represent those of their affiliated organizations, or those of the publisher, the editors and the reviewers. Any product that may be evaluated in this article, or claim that may be made by its manufacturer, is not guaranteed or endorsed by the publisher.

## Footnotes

1
https://www.researchgate.net/publication/281445479_Geoscience_2020_Cyberinfrastructure_to_reveal_the_past_comprehend_the_presentand_envision_the_future

2
https://frictionlessdata.io/

3
https://github.com/hurwitzlab/planet-microbe-datapackages

4
https://goodtables.readthedocs.io/en/latest/

5
https://github.com/hurwitzlab/planet-microbe-ontology

6
https://doi.org/10.5281/zenodo.4973944

7
https://oceanservice.noaa.gov/facts/thermocline.html

8
https://www.ncbi.nlm.nih.gov/biosample/SAMN06061931

9
https://github.com/hurwitzlab/planet-microbe-scripts

10
http://dx.doi.org/10.17504/protocols.io.bzsdp6a6

11
https://github.com/EnvironmentOntology/envo/blob/master/src/envo/modules/chemical_concentration.csv

12
https://www.microb3.eu/sites/default/files/osd/OSD_HandbooK_2016.pdf

13
https://genome-idx.s3.amazonaws.com/bt/GRCh38_noalt_as.zip

14
https://benlangmead.github.io/aws-indexes/k2

15
https://github.com/aponsero/readbased_metagenomes_snakemake

16
https://www.planetmicrobe.org/#/search

17
https://community.rstudio.com/t/correction-in-correlation-method-in-rquery-cormat/73031

18
https://inkscape.org/
