# Supplementary figures and images for: Ontology-Enriched Specifications Enabling Findable, Accessible, Interoperable, and Reusable Marine Metagenomic Datasets in Cyberinfrastructure Systems
Source: Front Microbiol. 2021 Dec 8;12:765268. doi: 10.3389/fmicb.2021.765268 (PMC8692764; doi:10.3389/fmicb.2021.765268)

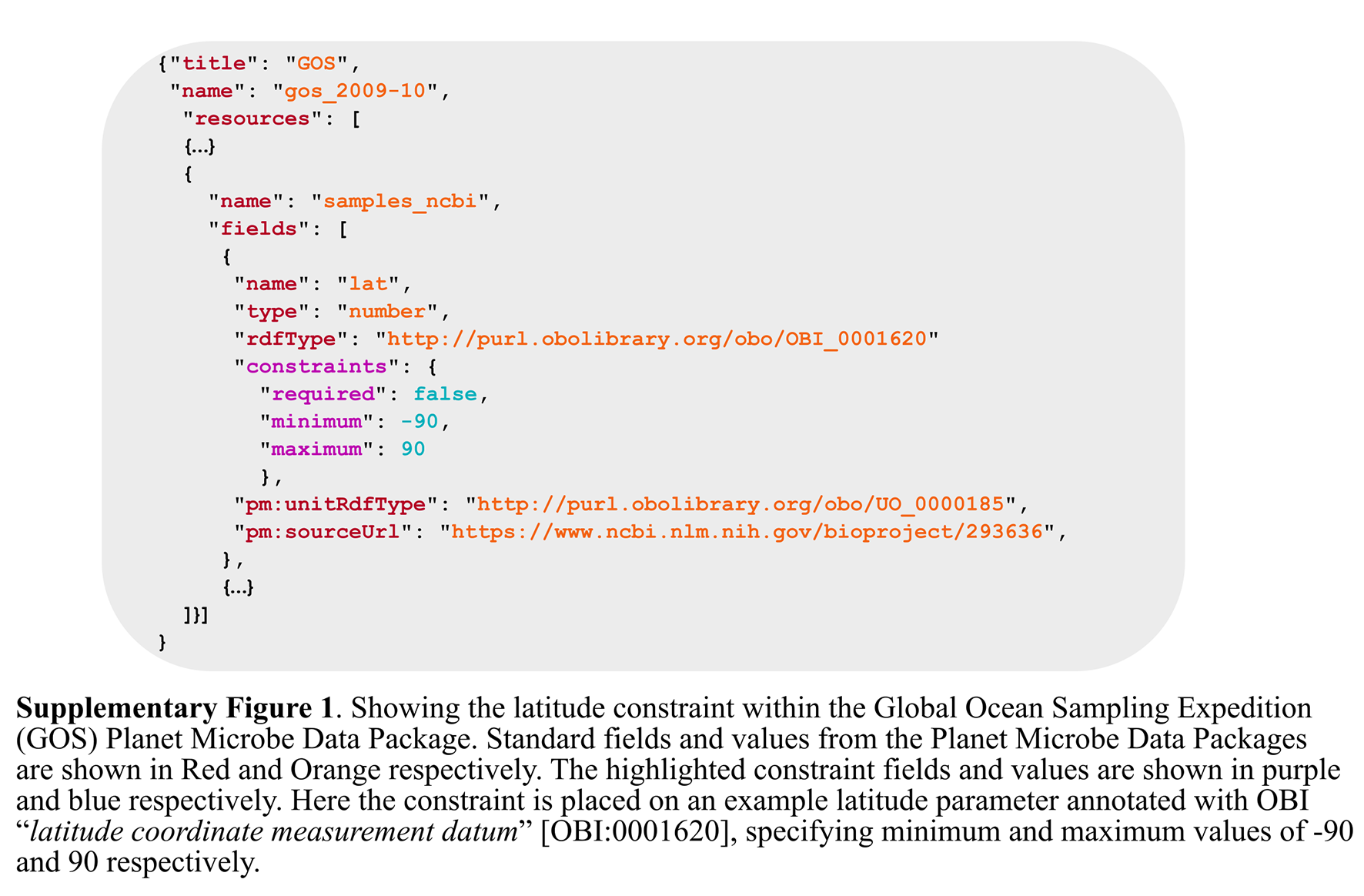

Supplement: Supplementary file 1 [file Image_1.TIFF]

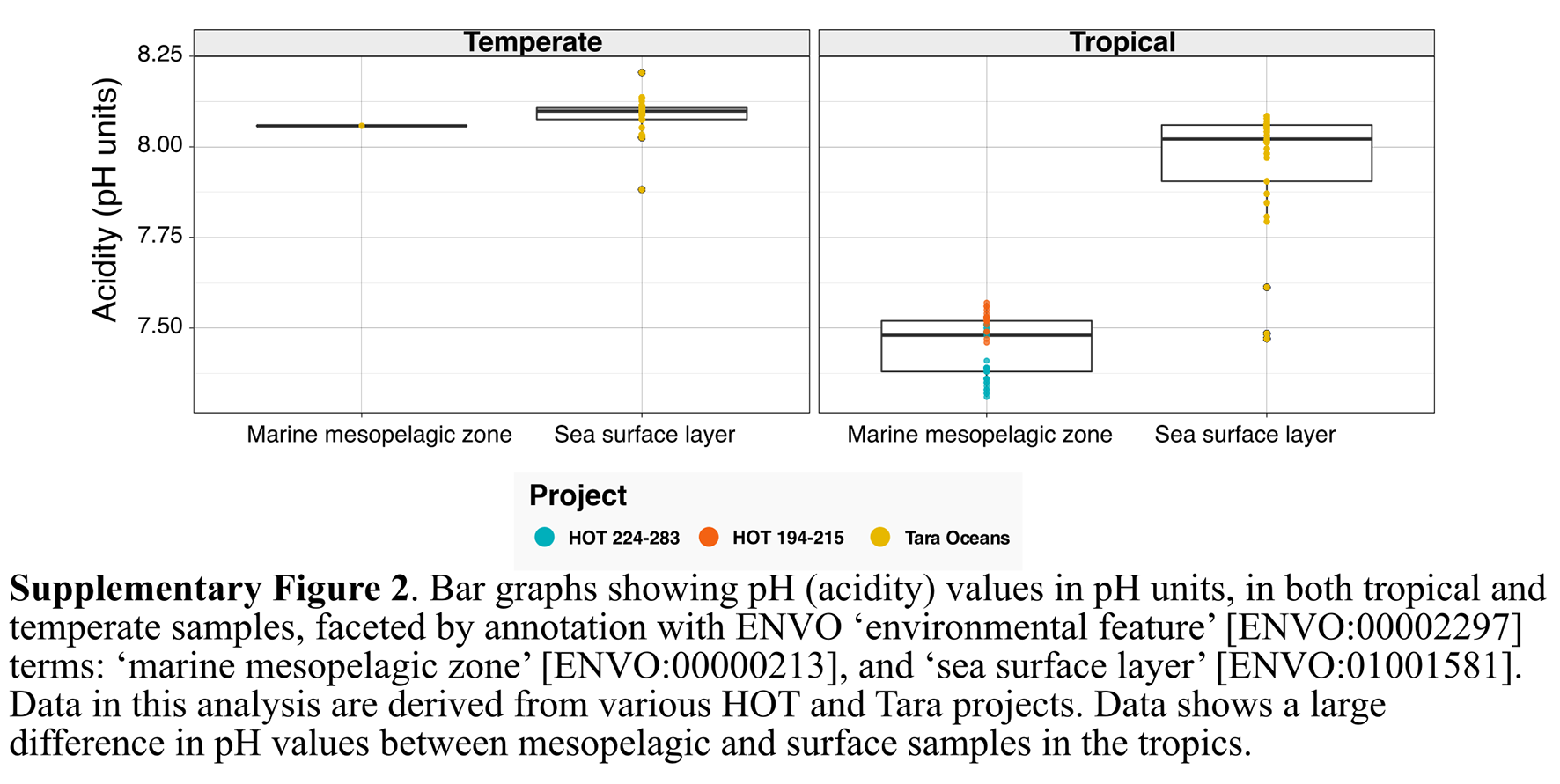

Supplement: Supplementary file 2 [file Image_2.tiff]

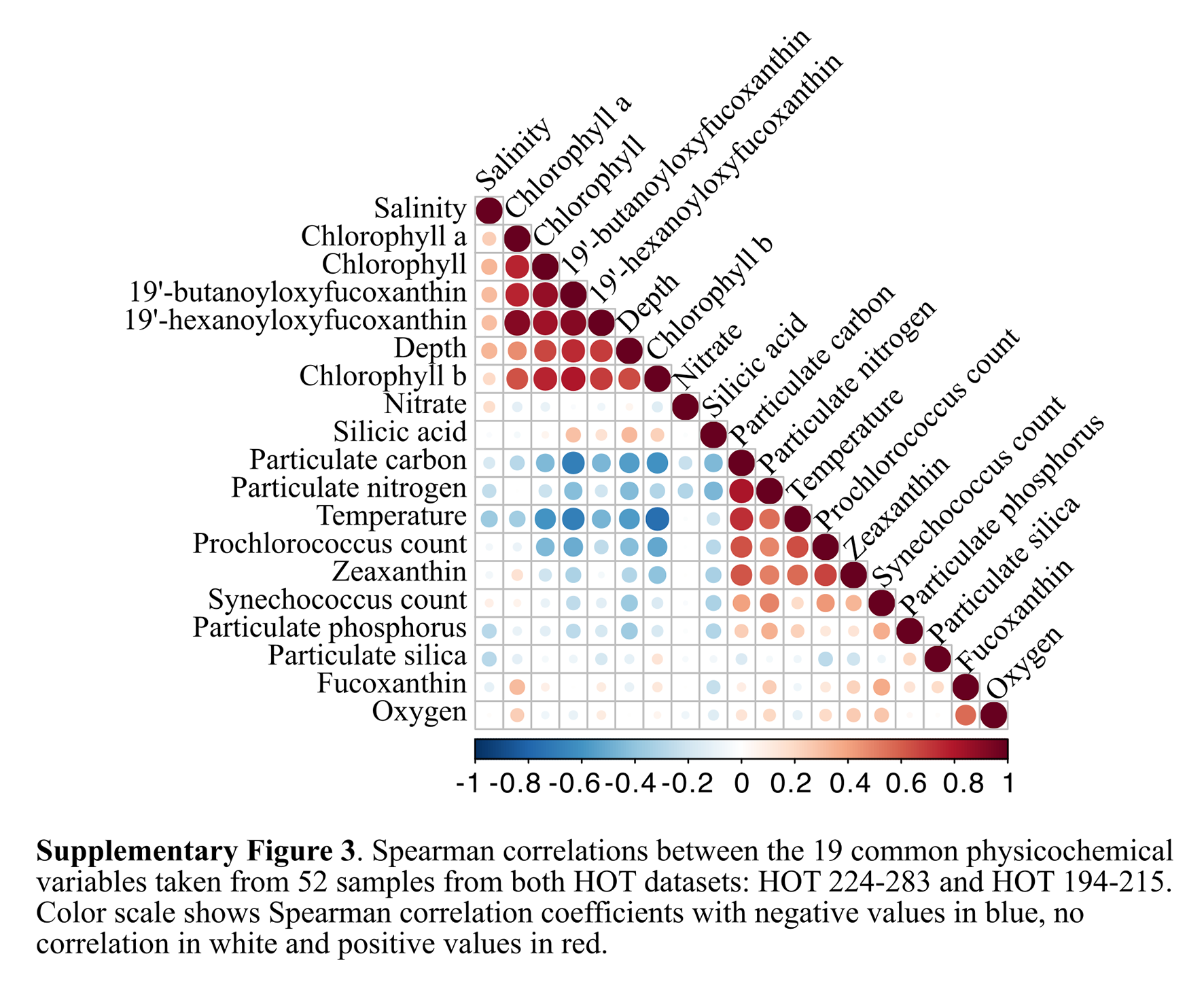

Supplement: Supplementary file 3 [file Image_3.TIFF]
